# Kingdom Come

**DOI:** 10.1371/journal.pgen.1009178

**Published:** 2020-11-05

**Authors:** Gregory S. Barsh, Gregory P. Copenhaver, Claudia Köhler, Li-Jia Qu

**Affiliations:** 1 HudsonAlpha Institute for Biotechnology, Huntsville, Alabama, United States of America; 2 Department of Genetics, Stanford University School of Medicine, Stanford, California, United States of America; 3 Department of Biology and the Integrative Program for Biological and Genome Sciences, University of North Carolina at Chapel Hill, Chapel Hill, North Carolina, United States of America; 4 Department of Plant Biology, Swedish University of Agricultural Sciences and Linnean Center for Plant Biology, Uppsala BioCenter, Uppsala, Sweden; 5 State Key Laboratory for Protein and Plant Gene Research, Peking-Tsinghua Center for Life Sciences at College of Life Sciences, Peking University, Beijing, China

**Fig 1:**
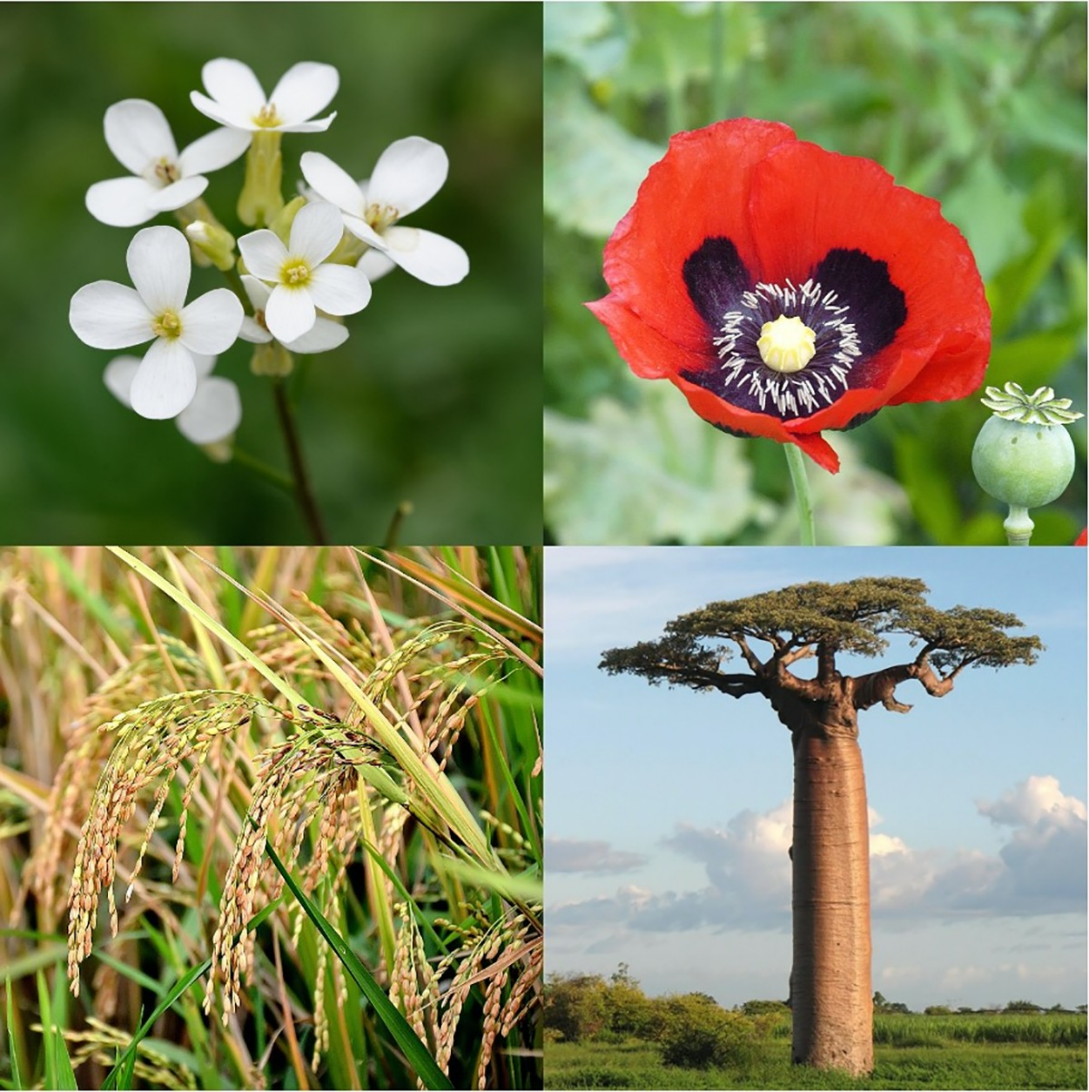
From top left to bottom right: the model flowering plant *Arabidopsis thaliana* (*Image credit*: *Marie-Lan Nguyen*, *Wikimedia Commons*, *CC-BY 2*.*5*), the opium poppy *Papaver somniferum* (*Image credit*: *Tanja Niggendijker*, *Wikimedia Commons*, *CC BY 2*.*0*), the Asian rice *Oryza sativa* (*Image credit*: *Augustus Binu*, *Wikimedia Commons*, *CC BY-SA 3*.*0*), and the Madagascar baobab tree *Adansonia grandidieri* (*Image credit*: *Bernard Gagnon*, *Wikimedia Commons*, *CC BY-SA 3*.*0*).

The tumultuous events of 2020 have prompted many of us to reflect upon the forces that divide us, like pandemics and politics, as well as the commonalities that unite us, like our shared hope for a better future. Scientists often face a similar problem—when to lump or split the things they study. *PLOS Genetics* has decided that while plants (Fig 1) obey the same fundamental genetic principles as other organisms, their unique qualities, many of which are critically important to human health and welfare, merit special attention in the same way as our other sections: Cancer Genetics, Epigenetics, Evolution, Methods, Natural Variation, and Prokaryotic Genetics. To support this goal, we are creating a new Plant Genetics section for the journal that will be led by the inaugural Senior Editors Claudia Köhler and Li-Jia Qu.

Plants are united with all other forms of life by fundamental aspects of biology, including relying on nucleic acids to store and transmit their genetic information. Nonetheless, recognizing the distinction between plants and animals (and more recently, bacteria, archaebacteria, protists, fungi, and chromista) is as old as written history, as codified by Carl Linnaeus in 1735 when he split the natural world into *Regnum Vegetabile*, *Regnum Animale*, and *Regnum Lapideum* (plants, animals, and minerals) in his book *Systema Naturae*. While there is still discussion about the arrangement, or taxonomy, of the various groupings of living things, plants are recognized as forming one of the broadest groups—a kingdom of life.

We study animals and microbial life because of our insatiable curiosity to understand how the natural world works and because of their immediate practical influence on our lives including identifying the agents that make us sick and developing the medicines and techniques that keep us healthy. Plants are equal partners in those missions. They are a quintessential part of our lived experience providing, either as a primary or secondary source, all of the food we consume, a significant portion of the energy we use, and the basis for much of our critical pharmacopeia. In addition, through their aesthetic qualities, plants contribute to our mental well-being—put simply, plants possess remarkable beauty and make us feel better by their mere presence.

We are lucky then that a legion of plant geneticists exists to help us better understand these useful and beautiful organisms. From deciphering how plants convert sunlight into stored biomolecular energy, delineating the pathways that produce a dazzling array of useful secondary metabolites, to engineering plants that produce lifesaving drugs and medicines, our new Plant Genetics section will be a home for the science that captures the leading edge of this exciting area. We welcome your patronage as readers and wholeheartedly encourage your participation as authors.

